# Supplementary material for: Gamma-ray Irradiation of Rodent Diets Alters the Urinary Metabolome in Rats with Chemically Induced Mammary Cancer
Source: Metabolites. 2022 Oct 16;12(10):976. doi: 10.3390/metabo12100976 (PMC9608802; doi:10.3390/metabo12100976)
Supplement: Supplementary file 1 [file metabolites-12-00976-s001.zip › metabolites-1951949-Supplementary Figures.pdf]

Gamma-ray irradiation of rodent diets alters the urinary metabolome in chemically induced mammary cancer in rats

Jeevan K. Prasain*[1], Landon S Wilson[3], Clinton Grubbs[2], Stephen Barnes[1,3]

Departments of Pharmacology and Toxicology[1] and Surgery[2], and Targeted Metabolomics and Proteomics Laboratory[3], University of Alabama at Birmingham, Birmingham, AL 35294

[A] [B]

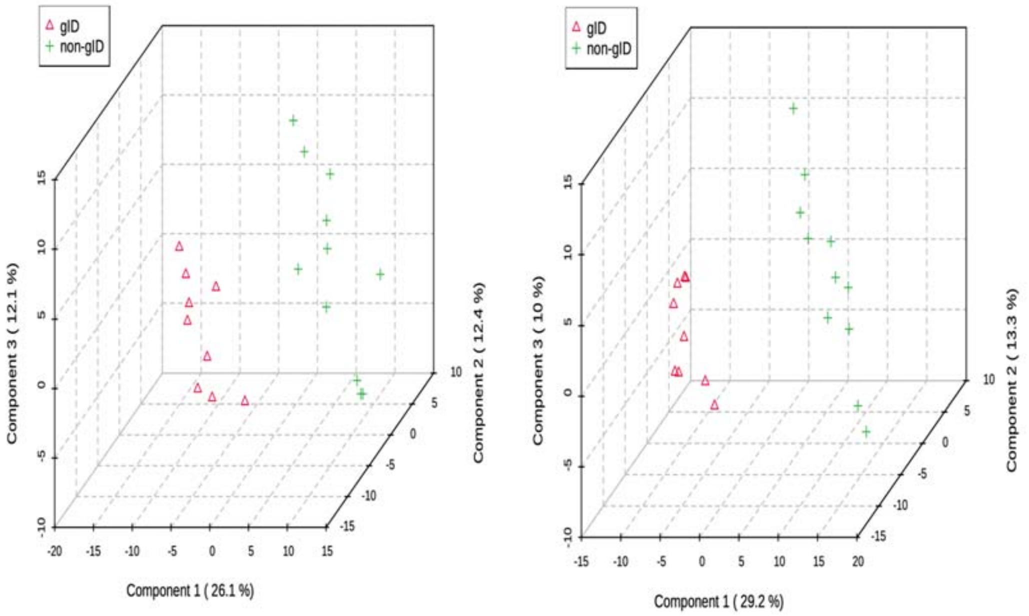

Figure S1. 3D-PLSDA score plots of urine samples from gID animals, non-gID fed animals. This analysis shows that urines from rats on gID and non-gID were completely separated in both negative [A] and positive [B] ion modes.

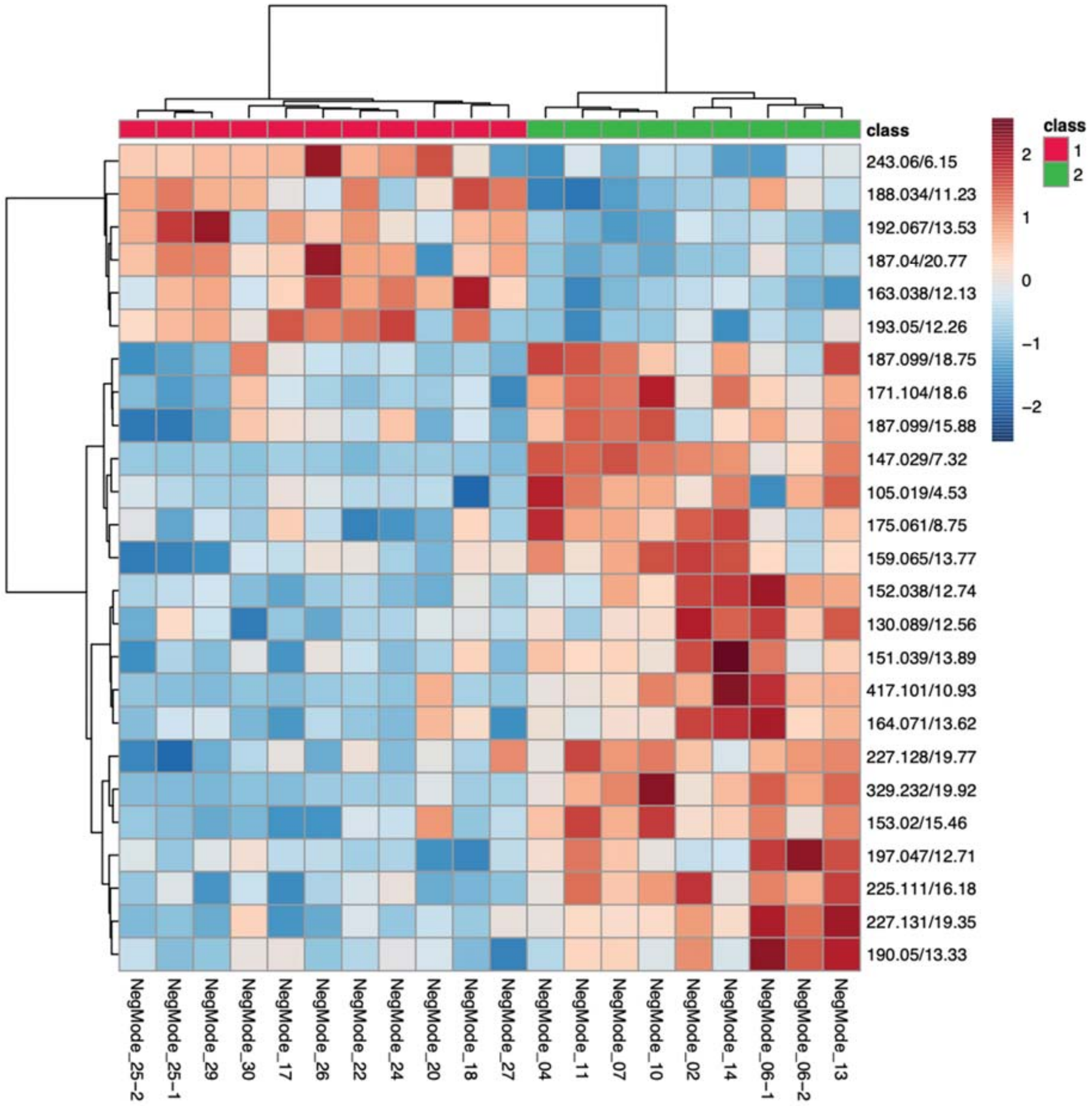

Figure S2. Heat maps of negative ion data from rat urines. This analysis shows that rats on gID and non-ID diets were completely separated. Candidate ions leading to the separation are presented. Changes largely consist of increases observed in urines of rats on the gID diet.

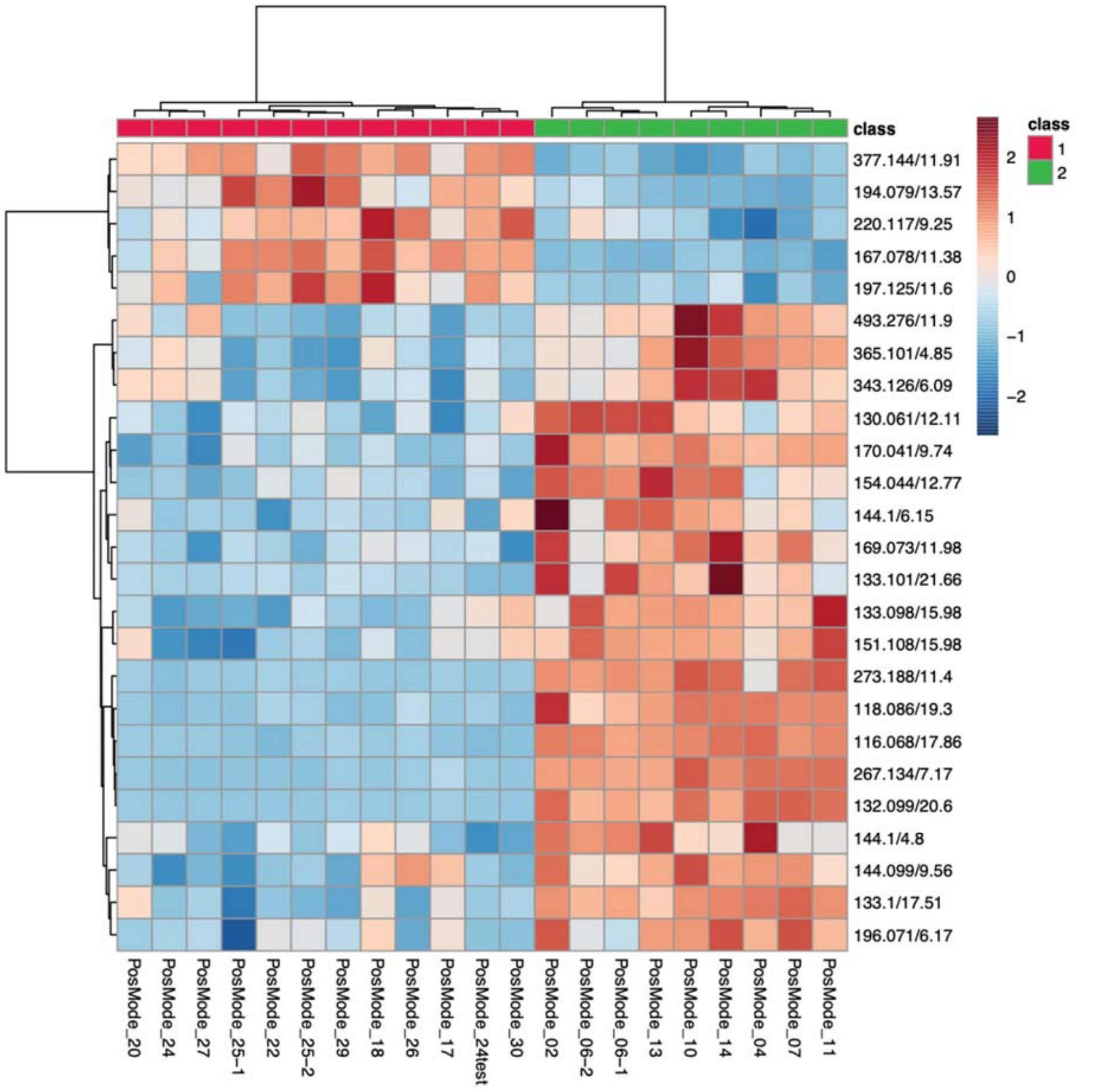

Figure S3. Heat maps of positive ion data from rat urines. This analysis shows that rats on gID and non-ID diets were completely separated. Candidate ions leading to the separation are presented. Changes largely consist of increases observed in urines of rats on the gID diet.

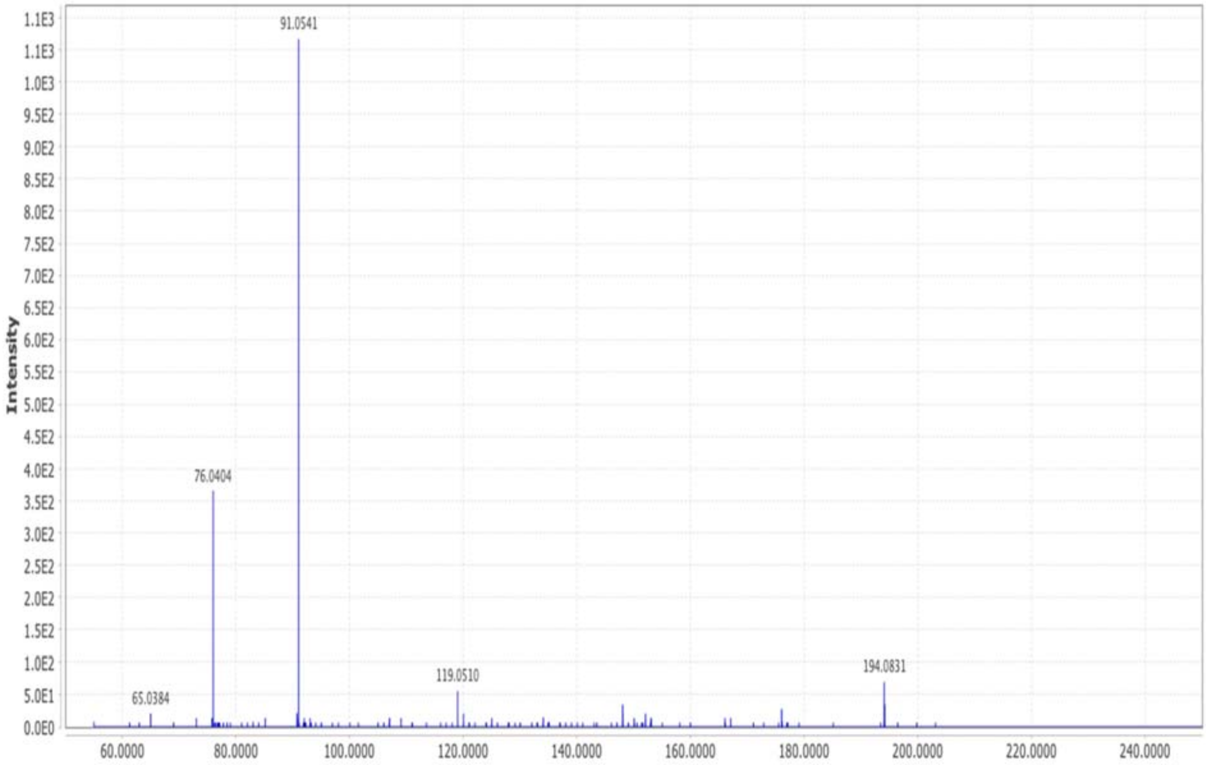

Figure S4. LC-MS/MS product ion spectrum of *m/z* 194.083 $[M+H]^+$, Rt 13.5 min.

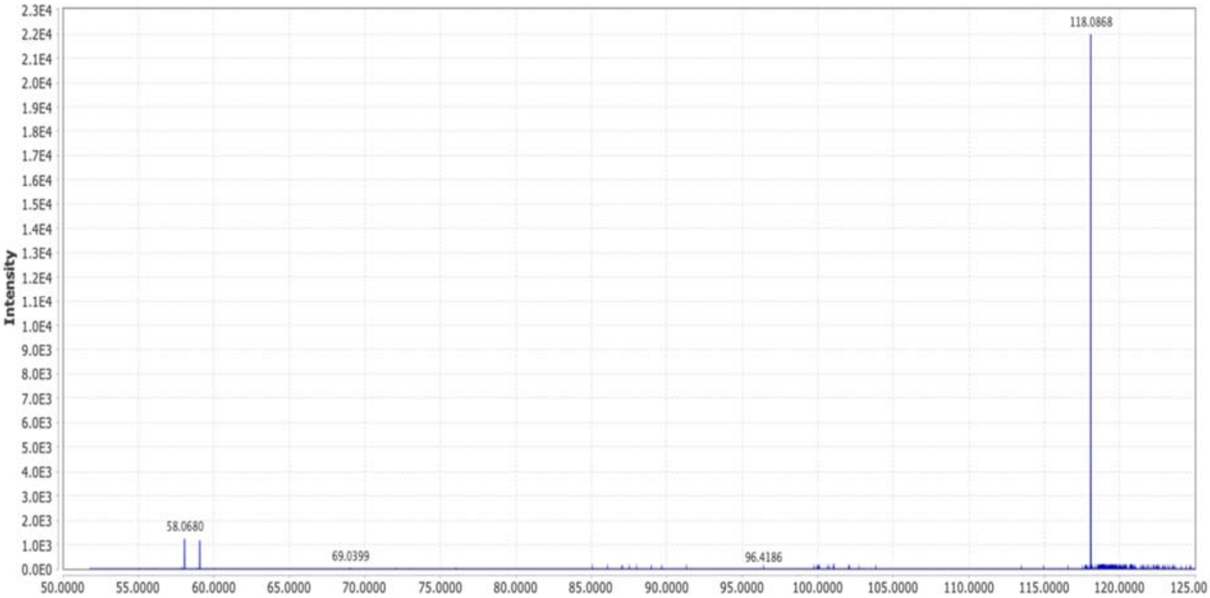

Figure S5. LC-MS/MS product ion spectrum of *m*/*z* 118.083 $[M+H]^+$, Rt 4.3 min.

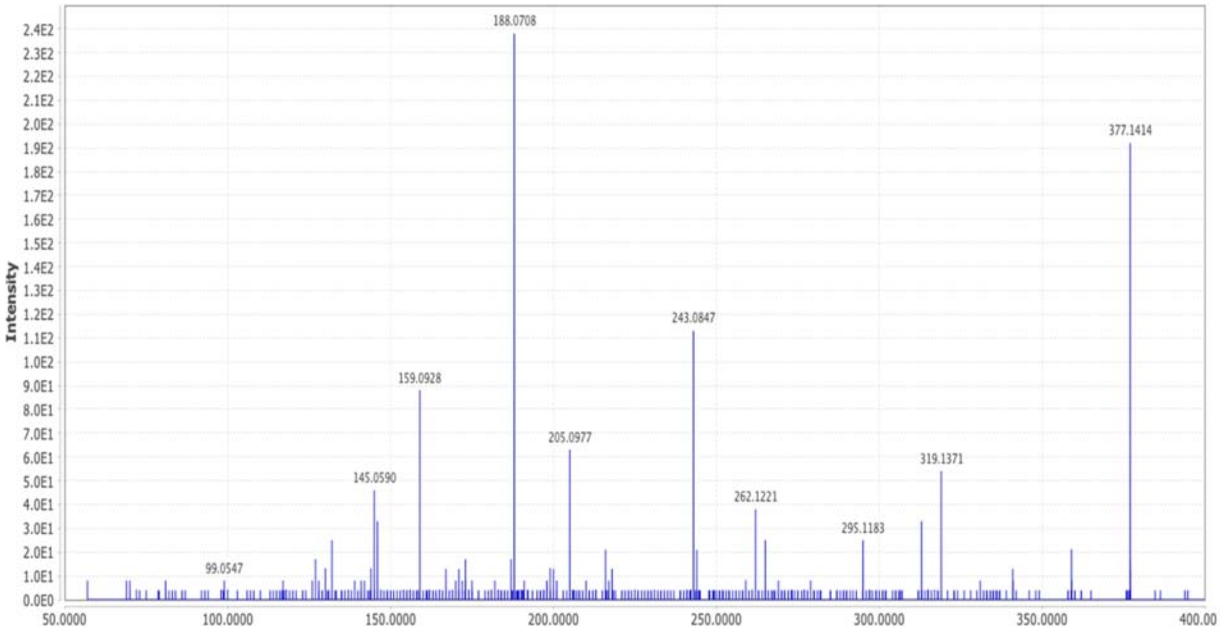

Figure S6. Product ion spectrum of *m*/*z* 377.146 $[M+H]^+$ showing an intense product ions *m*/*z* 243.087 indicated this metabolite to be riboflavin vitamin B2 (cal. *m*/*z* 377.145).

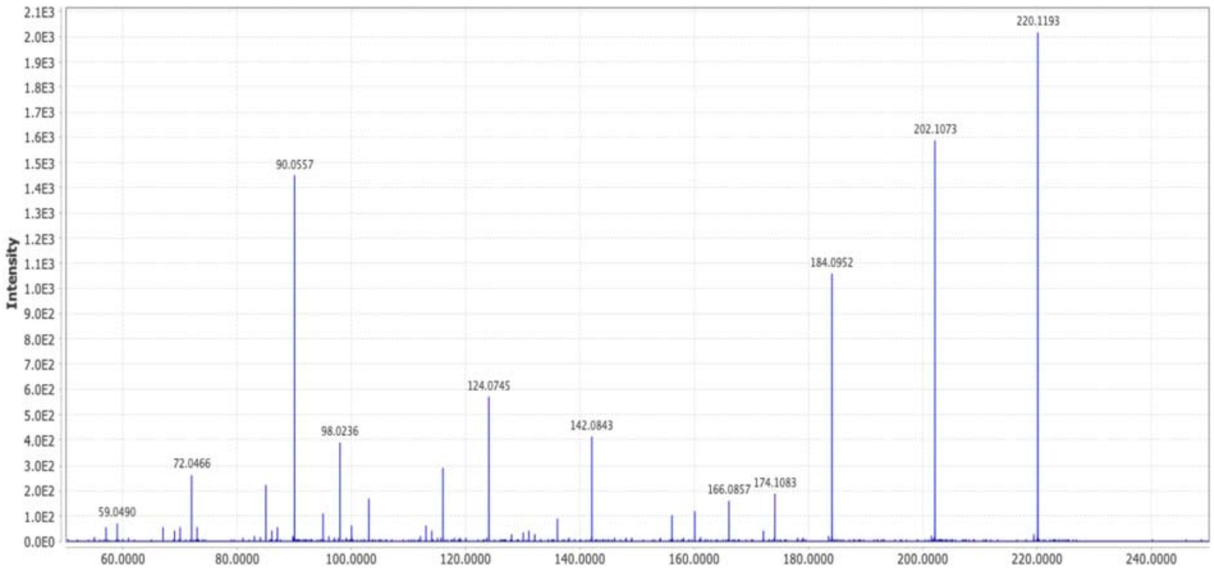

Figure S7. Product ion spectrum of *m*/*z* 220.117 [M+H]$^+$, Rt 9.2 min.

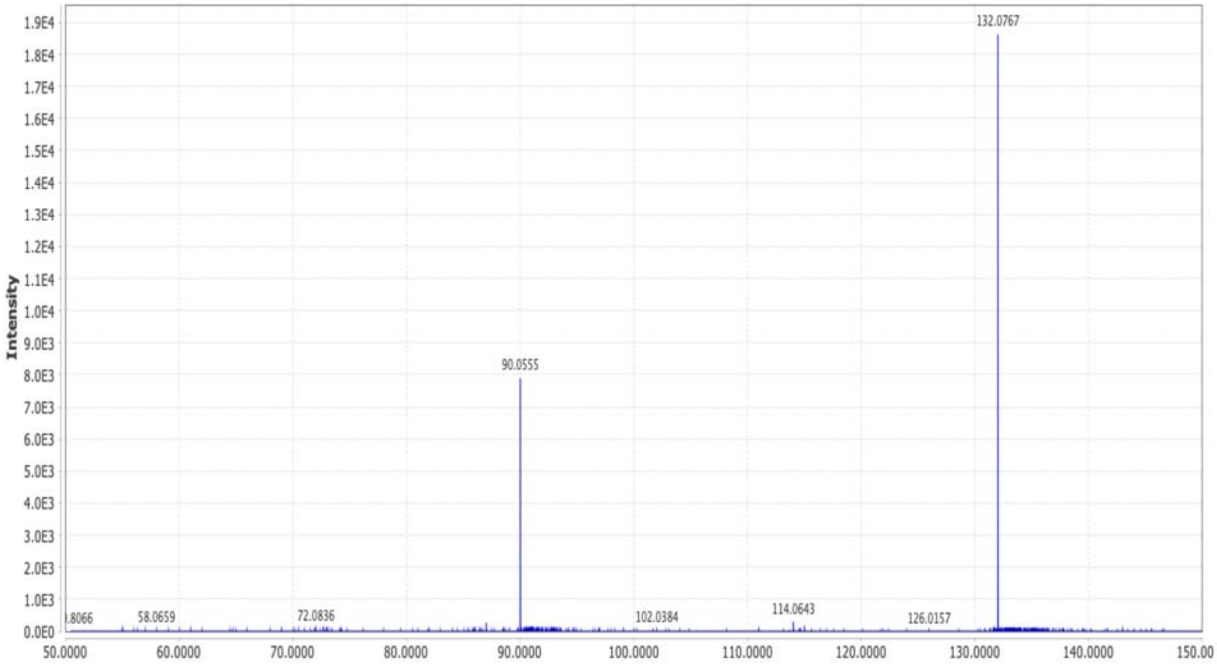

Figure S8. Product ion spectrum of *m*/*z* 132.074 [M+H]$^+$, Rt 4.4 min.

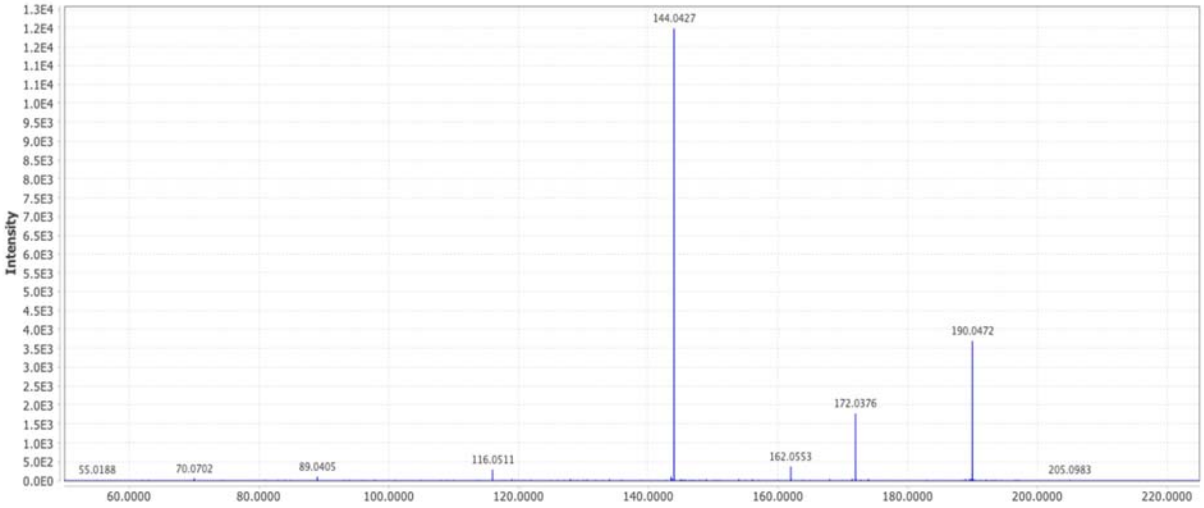

Figure S9. LC-MS/MS product ion spectrum of *m/z* 190.047 [M+H]+, Rt 11.2 min.

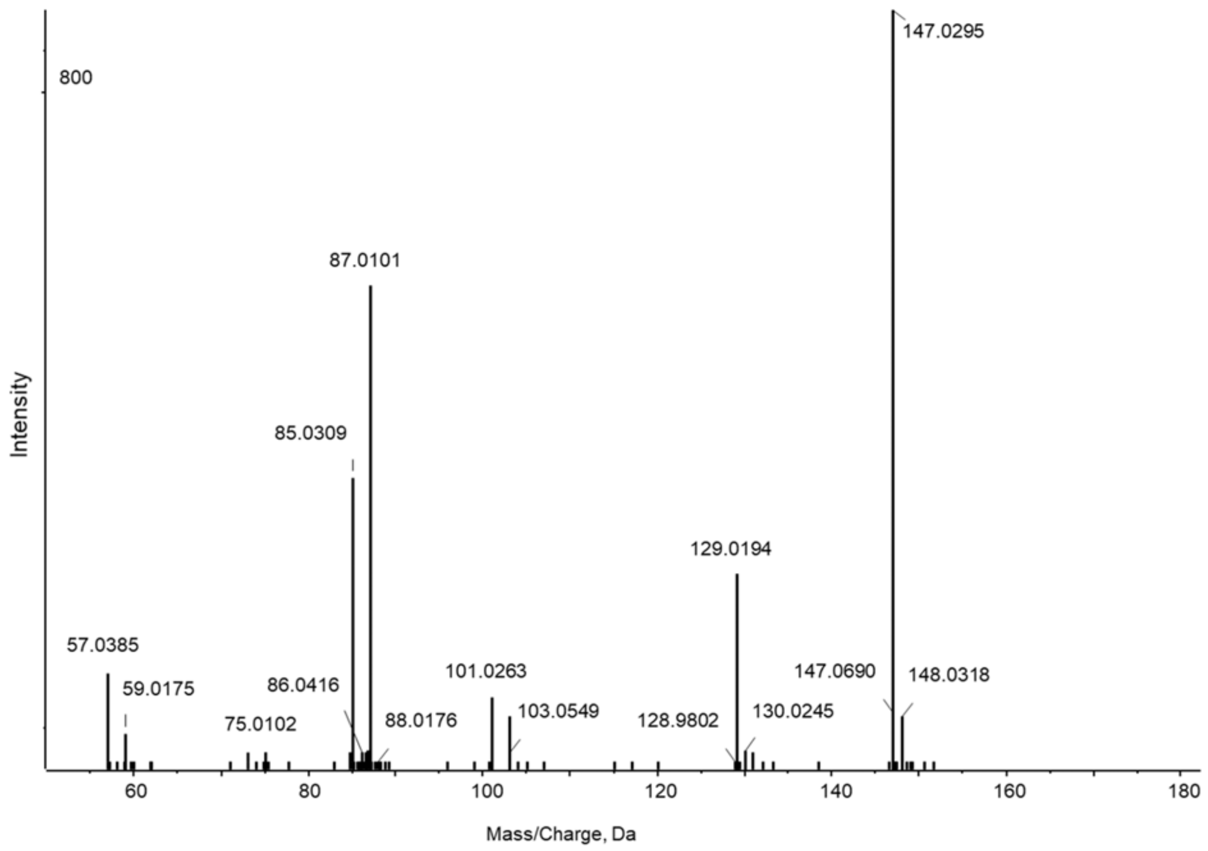

Figure S10. Product ion spectrum of *m/z* 147.029 [M-H]- showing intense product ions *m/z* 129.012 together with 87.010 and 85.030 indicated this metabolite to be citramalate (cal. m/z 147.029)
