# Supplementary material for: Gamma-ray Irradiation of Rodent Diets Alters the Urinary Metabolome in Rats with Chemically Induced Mammary Cancer
Source: Metabolites. 2022 Oct 16;12(10):976. doi: 10.3390/metabo12100976 (PMC9608802; doi:10.3390/metabo12100976)
Supplement: Supplementary file 1 [file metabolites-12-00976-s001.zip › metabolites-1951949-Supplementary Table.pdf]

Gamma-ray irradiation of rodent diets alters the urinary metabolome in chemically induced mammary cancer in rats

Jeevan K. Prasain*1, Landon S Wilson3, Clinton Grubbs2, Stephen Barnes1,3
Departments of Pharmacology and Toxicology1 and Surgery2, and Targeted Metabolomics and Proteomics Laboratory3, University of Alabama at Birmingham, Birmingham, AL 35294

Table S1. Mummichog pathway analysis of negative and positive ion data.

**Negative ion data**			
Pathways	overlap_size	pathway _size	p-value
Butanoate metabolism	17	19	0.0064
Linoleate metabolism	13	16	0.0092
Lysine metabolism	18	24	0.0092
Drug metabolism-Cytochrome p450	24	35	0.0115
Valine, leucine, and isoleucine degradation	18	25	0.0115
Tryptophan metabolism	38	59	0.0119
Heparan sulfate degradation	5	5	0.0207
Chondroitin sulfate degradation	5	5	0.020
N-glycan degradation	6	7	0.275
Fatty acid metabolism	6	7	0.275
Keratin sulfate degradation	6	7	0.275
**Positive ion data**			
Pathways	overlap_size	pathway _size	p-value
Drug metabolism-Cytochrome p450	20	35	0.0056
Lysine metabolism	9	14	0.0092
Fatty acid metabolism	4	5	0.0218
Methionine and cysteine metabolism	13	25	0.0263
Histidine metabolism	6	10	0.0348
Purine metabolism	17	35	0.0389
